# A conversation with Jason Abaluck, Professor, Economics, Yale University School of Management

**DOI:** 10.1017/cts.2023.710

**Published:** 2024-01-17

**Authors:** Clinical Research Forum

**Affiliations:** Clinical Research Forum, Washington, DC, USA

## Top 10 Clinical Research Achievement Awards Q & A

This article is part of a series of interviews with recipients of Clinical Research Forum’s Top 10 Clinical Research Achievement Awards. This article is about Dr Jason Abaluck, Professor, Economics, Yale University School of Management. Dr Abaluck’s research focuses on the detection of mistakes and the design of institutions when consumers or providers make mistakes in contexts such as health plan choice, dietary choice, or the provision of medical care. He received a 2023 Top 10 Clinical Research Achievement Award for *Impact of community masking on COVID-19: A cluster-randomized trial in Bangladesh. The interview has been edited for length and clarity.*

## What do you like most about working in the field of economics?

I like thinking about things that have an impact on the world and economics is the most mathematical social science for thinking systematically about those things. Economics differs from the other social sciences in that we try to arrive at bottom-line answers, albeit provisional ones. Like others, we ask questions like: what are the things I care about in the world? What can be done about those things? But we also care about saying: what are the costs and benefits of our actions? What is the best decision we can make given the information available to us now? I’m interested in learning what kind of evidence should be gathered to better make those decisions and, more generally, what’s the most constructive thing that can be done at any given time to help us make better decisions.

## When did you start thinking about these questions in relation to human health and health policies?

That’s something that I came to in graduate school. If you’re trying to determine how you can help improve people’s lives, it turns out that keeping them healthy is one of the most important things you can do. My perspective as an economist is different than epidemiologists and other health researchers. For me, the focus is on identifying and measuring the costs and the benefits of the behaviors or policies we’re studying. These days, most of my work is in the realm of quantifying the benefits and costs of health policy.

## So, during the pandemic, you became curious about the benefits and costs of masking?

Yes, back in March 2020, COVID was raising many policy issues, and a colleague of mine happened to ask me what I thought about the literature around masking. That’s when I started looking into it. At the time there weren’t many papers about masking, and the studies that had been done were missing one very critical element: they didn’t observe whether people were actually wearing masks. The researchers would ask participants to wear a mask, and then they would ask them later if they wore a mask. But there’s a big problem with this approach. When you ask someone to wear a mask, and then you ask them if they wore a mask, they’re probably going to say yes regardless of whether they did so. Now we have a huge amount of evidence that this is the case. So, since these earlier studies didn’t know who was actually wearing a mask, if the results show no impact, there are two interpretations. One interpretation is that masks don’t work. The second interpretation is you didn’t succeed in getting people to wear a mask. That’s why you want to do a trial like the one we did.

## What did the award-winning trial show?

Across 600 villages in Bangladesh, we randomized 300 of them to have an intervention that was designed to increase mask wearing. What we found was that in the villages that had interventions designed to increase mask wearing, the rates of COVID were lower by about 10%. Does this mean that masks reduce COVID by 10%? Well, not quite, because the first question you have to ask is the question I asked about the previous studies which is, how much did mask wearing increase? It turns out we were able to increase mask wearing by about 30 percentage points. That means the headline result of the study is essentially a 30 percentage point increase in mask wearing reduces COVID infections by about 10%. If you could increase mask wearing even more, you might expect that would reduce infections by even more.

## What kind of interventions were tried to increase mask wearing?

We gave people information about why mask wearing is important, and we gave them masks. In addition, there was a person in the community who went around and observed whether people were wearing masks and if they weren’t, they would ask them to please, put on a mask. We also had community leaders wearing masks and modeling behaviors. It was the combination of all of these things that increased mask wearing by about 30%. This is not saying this is easy to do. But this is what earlier studies didn’t do, which makes me think they probably didn’t increase mask wearing. Other studies look at what happens when you give people only free masks or information, and those find much smaller impacts on mask wearing than our study.

## How does an individual’s perception of risk factor into this type of cost–benefit analysis?

It’s transparently clear when you’re doing these calculations that the value of mask wearing was going to change with the underlying circumstances. For instance, if you’re in the world where there’s a respiratory virus and no vaccinations, then the value of mask wearing is tremendously high. If vaccinations become available, at least in some places for some people, the risk is diminished – again, for some people – and the calculations get more complicated. You have to think about the number of people vaccinated, the impacts of the different variants of COVID – all of those kinds of things are going to change the answer for the value of mask wearing at different places and times and for different people.

## This study required collaboration across multiple disciplines, particularly between epidemiologists and economists. Did that create challenges? If so, how did you overcome them?

Economists are very familiar with running these kinds of large-scale field experiments, but they’re done much less commonly in medicine and other social sciences. That’s a deficiency, I think, and I’d like to see those disciplines doing more big field experiments to figure out the impact of policies. Even so, I’d say that designing and running the trial – determining what needed to be done and the right ways to do it – was relatively smooth. We all recognized that people on the team had different specialties and would be responsible for different elements. The trickier part came when it was time to write the paper. There are differences across fields about how results are reported. It’s not that one or the other is correct. It’s just that in economics, statistics is fundamental to what we do, and we tend to speak and write papers with the assumption that readers are very knowledgeable about these things. Someone else might feel like there’s a way to present the results that helps others with a different background better understand what’s going on. There are trade-offs and we all learned from one another.

## Is this research continuing?

We’re exploring follow-up studies, not necessarily with COVID but with other respiratory infections and how they are spread in real-world situations. For instance, we’d like to look at how respiratory infections spread in school settings. That would help us answer the question: If a person who’s coughing and sneezing wears a mask at school, does that result in fewer respiratory infections?

## What other studies are you working on?

I’m really excited about some new research that is looking at how GPT-4 can improve patient outcomes. Basically, my view is that every clinician in the world can benefit from the advances in this technology. It used to be that to get algorithmic support for a health provider, the way the information was recorded had to be standardized. But with GPT-4, you don’t need to do that. Let’s say a patient has a rash and goes to see their doctor. The doctor can take a photo or record an audio conversation and upload it on an app. Then GPT4 can analyze it and, if necessary, come back with, “Hey, here’s something you might have missed.” So you get a second opinion without having to go see another clinician. We’re running a trial in Nigeria to see if using GPT4 in this way improves outcomes.

## What hobbies do you pursue in your “downtime” and how do they impact your research?

I like playing table tennis and I like playing video games. I would say the main way they impact my research is if I’m playing table tennis or a video game, then I’m not doing my research. *(Laughter.)*

